# The Engineering Side of Hospital Work

**Published:** 1905-05-06

**Authors:** 


### HOSPITAL ADMINISTRATION.

### CONSTRUCTION AND ECONOMICS.

### THE ENGINEERING SIDE OF HOSPITAL WORK.

(*Continued from page* 94)

XI.—LAUNDRIES.

Of the hospitals visited by the writer, St. George's, Charing Cross, Westminster, and St. Thomas's, have no laundry.

Middlesex Hospital.

Middlesex has a laundry capable of doing 30,000 pieces a week, and now doing 16,000, at Hendon, the clothes being placed in bags when going to the laundry, and in baskets when returning, and stacked in a box-waggon. The waggon is one of Thornycroft's steam lorries arranged for the purpose. The steam lorry makes one complete journey daily to and fro, and the writer was informed by the engineer that it had saved £100 per annum over horse haulage. The roads leading to Hendon were very bad last summer, owing to repairs and the usual disturbances that Londoners are so familiar with, but though horse traffic had been obliged to be stopped, the motor-van had never stopped. The plant at Hendon comprises rotary washing machines, and centrifugal drying apparatus made by Messrs. Manlove, Alliott, and Co. The drying is carried on in air-drying rooms, not in closets. The clothes are hung on lines in the rooms, and air warmed by Sturtevant's apparatus is forced into the room by a fan, is allowed to pass into the washhouse from the drying-room, and is drawn out from the top of the washhouse by another fan.

St. Bartholomew's Hospital.

St. Bartholomew's has a laundry at Swanley, washing 14,000 articles a week. It has four rotary washing-machines, two centrifugal wringers, and a drying-closet with 24 draw-out horses, the closets being heated by steam-pipes underneath, air being forced up over the steam pipes, over the clothes, and out by the flue. They have also a large open-air drying ground. Clothes are taken down by Pickford & Co. in specially closed vans. The clothes are taken down in bags, and returned in bags and baskets. The van goes down one day and returns the next. Loads of three tons are taken at each journey. The laundry and drying-ground at Swanley are in connection with the hospital's Convalescent Home at Swanley.

The London Hospital Laundry.

The London Hospital has a very complete laundry. It is divided into two parts, the patients' clothes being dealt with in one part and the nurses' in the other. In all 50,000 pieces of clothes are dealt with per week. In the washing room of the nurses' portion, there are three Bradford's washing machines, and five boiling pans, these being very similar to the old-time copper, the heating being done by passing steam into the pans, in the same way as it is passed into the washing machines. There are also two 42-inch centrifugal wringing machines, one wringing and wrincing machine, one starch boiler, and one small flannel washer. In the patients' portion there are five washing machines, of the capacity of 200 shirts each, one of them being used for foul linen, two 42-inch centrifugal wringing machines, and three boiling pans.

In the finishing department, on the nurses' side there are one steam-heated cylinder ironing machine, and three of Bradford's Vesta skirt ironing machines. In the patients' portion of the finishing department there is one large Decoudun ironing machine, made by Bradford, having two rollers, steam heated, the rollers being nine feet long, and three women being employed on each side of the machine. There is also a six feet cylinder ironing machine for smaller things, and a mangle. For the hand-ironing room there are two iron holding towers each holding 50 irons.

The drying is accomplished principally by a 40-horse drying closet, of the form described in the article on drying apparatus. The apparatus was put in by Messrs. Bradford, and the horses run in and out of the drying closet on rollers and rails. The heating of the air is by means of two boxes of pipes, through which steam from the exhaust of the engine driving the laundry passes. The air is caused to pass over the steam pipes by two 3-foot fans, driven by two 4½ horse-power electric motors. The air is taken from the ironing room, and, after passing over the steam coils and through the clothes on the horses, is driven out into the flannel drying room and thence into the washing room, from which it is exhausted by a fan in the roof, carrying the steam from the washing machines with it. There is also an airing room, where the clothes are finally placed before being sent to the wards, the airing room being heated by two steam radiators.

There are also sorting rooms for the nurses' and the patients' clothes, where each nurse's things are taken to her pigeon-hole, etc.

The laundry is driven by a single cylinder engine of 40 horse-power, the power being delivered to the different machines by means of shafting and belts. It is intended to substitute an electric motor for the engine later on. The engine is supplied with steam from the boilers, 600 feet away, the drop in the steam-pressure between the boilers and the laundry being only from 63 lb. per square inch to 60 lb. per square inch. As mentioned above, the exhaust-steam from the engine is employed to heat the air for the drying closets.

Guy's Hospital.

Some of the clothes from Charing Cross Hospital are washed at Guy's, the total quantity dealt with being 35,000 pieces per week. There are four of Tullis' rotary washing machines, arranged specially for foul articles, a stream of water being kept running through the washing machine, which is closed the whole time, till all foulness has passed off. In addition there are nine Tullis' rotary washing-machines for the ordinary work. The Tullis machine is very similar to those described in the article on washing machines. There are eight centrifugal wringing machines, two of Tullis' manufacture, and six by Messrs. Summerscales. The washing machines are driven from shafting, which receives its power from a 10 horse-power electric motor, the eight centrifugal machines being driven by eight electric motors of 4 horse-power each. The drying is in a drying closet, with 42 horses in three sets. The electric motors all receive power from the hospital generating station. The air for the closets is heated by coils of pipes in which live steam from the boilers is passing, the pressure of the steam being reduced to 40 lb. for this work by a reducing valve arranged for the purpose. The air is taken from the engine room, which is just under the laundry, and, after passing through the closets, is passed out into the air at the side of the building. The air is propelled by three Sturtevant fans, each of 30 inches diameter, driven by electric motors of

1 horse-power each. In the finishing department there are two large cylinder ironing machines, each having four rollers, running in concave-shaped hollow iron beds, the beds being heated by steam. There are also two of Bradford's mangling machines, and four ironing machines, for skirts, etc., that are not much used, hand irons being preferred. The hand irons are heated by gas, the inside of the irons containing small gas stoves, or burners, arranged to burn the usual mixture of gas and air, as in the well-known Bunsen burner. There was no smell from the irons when the writer visited the ironing room.

St. Mary's Hospital.

At St. Mary's Hospital there are two of Bradford's rotary washing machines and one centrifugal wringing machine in the washing department, the whole being run by belts from shafting, the shafting receiving its power from a vertical engine having a 10-inch diameter cylinder with an 18-inch stroke. A mangle is also driven off the same shafting. The drying is by a closet, having nine horses heated by steam passing in 4-inch pipes under the horses. The air passes in under the front of the closets, through the clothes, and out at a shaft at the back, the shaft, or chimney, going right up to the roof, the column of hot air in the shaft providing the motive power for propelling the air through the closets. Ironing is by the ordinary hand irons, heated at a stove arranged for the purpose, the stove burning coke, the hot gases and smoke from the stove passing into the shaft of the drying closets, and assisting to create the necessary draught for the circulation of the air.

University Hospital.

At University Hospital there is one Bradford rotary washing machine, and one Bradford centrifugal wringing machine in the washing department. There is a mangle, and a drying closet with 12 horses of the usual pattern. There is a fan, which draws air from the laundry into the drying closets, forcing it over a nest of steam pipes, which are fixed over the horses, and then forcing it out through a flue to gratings leading to the outside of the building.

